# The Recruitment Innovation Center: Developing novel, person-centered strategies for clinical trial recruitment and retention

**DOI:** 10.1017/cts.2021.841

**Published:** 2021-08-19

**Authors:** Consuelo H. Wilkins, Terri L. Edwards, Mary Stroud, Nan Kennedy, Rebecca N. Jerome, Colleen E. Lawrence, Sheila V. Kusnoor, Sarah Nelson, Loretta M. Byrne, Leslie R. Boone, Julia Dunagan, Tiffany Israel, Casey Rodweller, Bethany Drury, Rhonda G. Kost, Jill M. Pulley, Gordon R. Bernard, Paul A. Harris

**Affiliations:** 1 Vanderbilt Institute for Clinical and Translational Research, Nashville, TN, USA; 2 Department of Medicine, Vanderbilt University Medical Center, Nashville, TN, USA; 3 Department of Internal Medicine, Meharry Medical College, Nashville, TN, USA; 4 Office of Health Equity, Vanderbilt University Medical Center, Nashville, TN, USA; 5 Center for Knowledge Management, Strategy and Innovation, Vanderbilt University Medical Center, Nashville, TN 37203 USA; 6 Center for Clinical and Translational Science, The Rockefeller University, New York, NY, USA; 7 Department of Biomedical Informatics, Vanderbilt University Medical Center, Nashville, TN, USA

**Keywords:** Translational research, multicenter clinical trials, participant recruitment, participant retention, CTSA program

## Abstract

Clinical trials continue to face significant challenges in participant recruitment and retention. The Recruitment Innovation Center (RIC), part of the Trial Innovation Network (TIN), has been funded by the National Center for Advancing Translational Sciences of the National Institutes of Health to develop innovative strategies and technologies to enhance participant engagement in all stages of multicenter clinical trials. In collaboration with investigator teams and liaisons at Clinical and Translational Science Award institutions, the RIC is charged with the mission to design, field-test, and refine novel resources in the context of individual clinical trials. These innovations are disseminated via newsletters, publications, a virtual toolbox on the TIN website, and RIC-hosted collaboration webinars. The RIC has designed, implemented, and promised customized recruitment support for 173 studies across many diverse disease areas. This support has incorporated site feasibility assessments, community input sessions, recruitment materials recommendations, social media campaigns, and an array of study-specific suggestions. The RIC’s goal is to evaluate the efficacy of these resources and provide access to all investigating teams, so that more trials can be completed on time, within budget, with diverse participation, and with enough accrual to power statistical analyses and make substantive contributions to the advancement of healthcare.

## Introduction

Difficulty in recruiting and retaining participants, long understood as a key research challenge, continues to plague the clinical trial landscape. Estimates suggest that nearly one in five studies either terminate for failed accrual or finish with underpowered numbers [1]. These issues not only add expense to research, but can also compromise generalizability and representativeness, raise ethical concerns, and defer the discovery of answers to significant health issues [2].

In recent years, increased attention to the adverse impact of recruitment shortfalls has led to identification of numerous contributing issues [3–7]. NIH’s National Center for Advancing Translational Sciences (NCATS) launched the Trial Innovation Network (TIN) in 2016 to create efficiencies, streamline processes, and discover and share solutions to common problems facing multicenter trial investigators. The TIN collaborative initiative includes three Trial Innovation Centers (TICs), one Recruitment Innovation Center (RIC), and the Clinical and Translational Science Awards (CTSA) Program Hubs [8,9]. Specific aims of the RIC are to:

1. Provide a national home and collaborative ‘storefront’ for the creation, storing, and sharing of recruitment education, programs, and best practices.
2. Catalyze enrollment by developing and disseminating novel technical and procedural approaches to support researchers in recruiting participants.
3. Enhance national awareness of research through patient education, and facilitate participant identification of studies with novel online patient facing tools.
4. Conduct rigorous studies on methods to enhance recruitment efficacy/efficiency and make modifications based on these data.

## The RIC Transdisciplinary Team

**Fig. 1.**
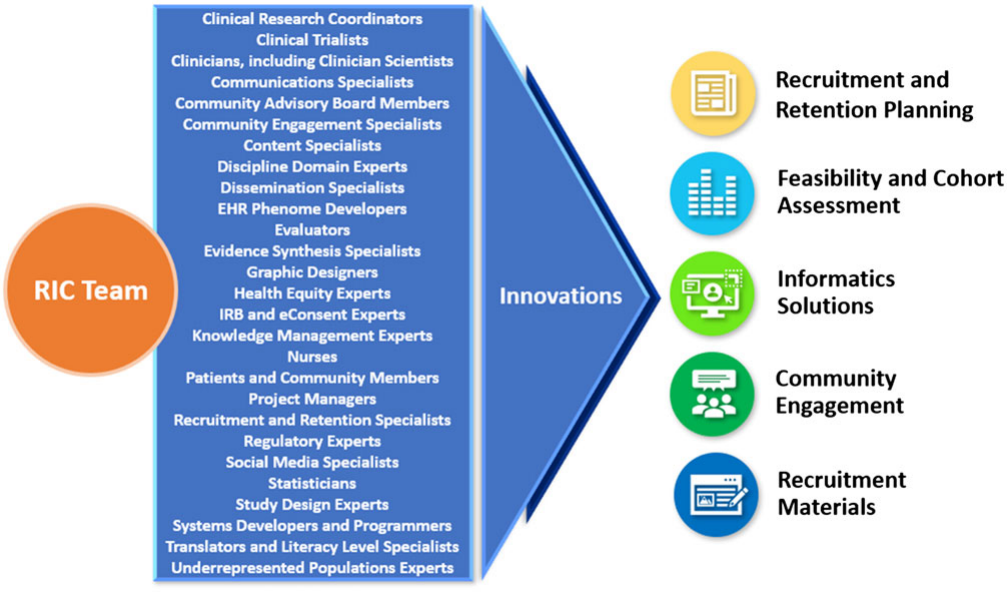
RIC transdisciplinary team of experts generates innovations in clinical trial recruitment and retention. RIC, Recruitment Innovation Center; IRB, Institutional Review Board; EHR, electronic health records.

Participant recruitment has evolved into a specialized field of knowledge requiring expertise from multiple stakeholders, including trialists, patients, front-line recruitment staff, advocacy groups, and community members. To achieve its aims, the RIC has assembled a transdisciplinary team of experts in diverse fields (Fig. 1). By tapping into the multiple perspectives and insights of this team, the RIC is empowered to design informatics-driven and community-engaged recruitment approaches across the continuum of research. With this holistic approach, the RIC develops custom strategies to optimize recruitment and retention for each study. The RIC team refines these potential solutions, disseminates them to the broader research community, then reconvenes in an iterative process to improve methods and explore new ground for innovation. This report describes the RIC’s unique team approach to resource design and delivery; details RIC resources; reports on progress and research team satisfaction metrics; and delineates plans to evaluate the effectiveness of its resources in improving clinical trial recruitment and retention.

## CTSA Engagement and the RIC Model for Design of Resources

The RIC’s resources are conceptualized and developed in parallel with trial teams to provide generalizable solutions to common recruitment challenges [2]. Typically, innovations are driven by and designed for a specific trial, and the resulting resources are tailored to the policies, guidelines, and cultures at participating CTSA Hubs. This accommodation of variability is a core principle of the RIC—every resource is designed to support generalizability while respecting local context. The local CTSA Points of Contact, who report to CTSA Principal Investigators (PIs) and Directors, are invited to participate in all consultations, enabling RIC consultants to determine what the local CTSA team can already provide to the PI.

Input from colleagues across the CTSA Network informed the RIC’s structure and resource offerings. In 2016 (Year 1 of the grant funding cycle), the RIC conducted a survey of CTSA PIs, Directors, and Recruitment Specialists at 53 responding institutions. Their ratings of the usefulness of forecasted RIC resources supported our choices for resource development (Supplemental Table 1). Sites also expressed a desire for the RIC to work directly with trialists to provide guidance on improving recruitment. Areas of recruitment cited as requiring extra support are listed in Supplemental Table 2.

Ongoing input from CTSAs is continuously collected through additional channels. Regularly scheduled TIN Collaboration Webinars enable CTSA Hub leaders and other team members to share their expertise, methods, and strategies in recruitment and retention. Monthly Open Forums and CTSA Liaison Team Meetings invite Network Project Leads and PIs to present on topics of importance to CTSA Hubs and provide an opportunity to share information and ask questions. The RIC also created and actively manages a Recruitment & Retention Working Group communications platform to support rapid online exchange of ideas, messaging, and discussions. This forum is used regularly by CTSA-wide experts to discuss current recruitment and retention topics.

The RIC’s Community Advisory Board (CAB), established in September 2017, also contributes to the creation, refinement, and evaluation of RIC-generated recruitment and engagement methods, as well as to the development of innovative practices. The CAB’s 12 members represent various geographic regions and diverse communities across the USA. They provide regular feedback that fosters a stronger, more productive relationship between the community and the research enterprise.

Additional rationale for developing RIC resources, as well as underlying hypotheses, resource allocation details, resource usage, and planned evaluation are documented in the RIC metrics and evaluation section.

## The Continuum of Research Recruitment Efforts

**Fig. 2.**
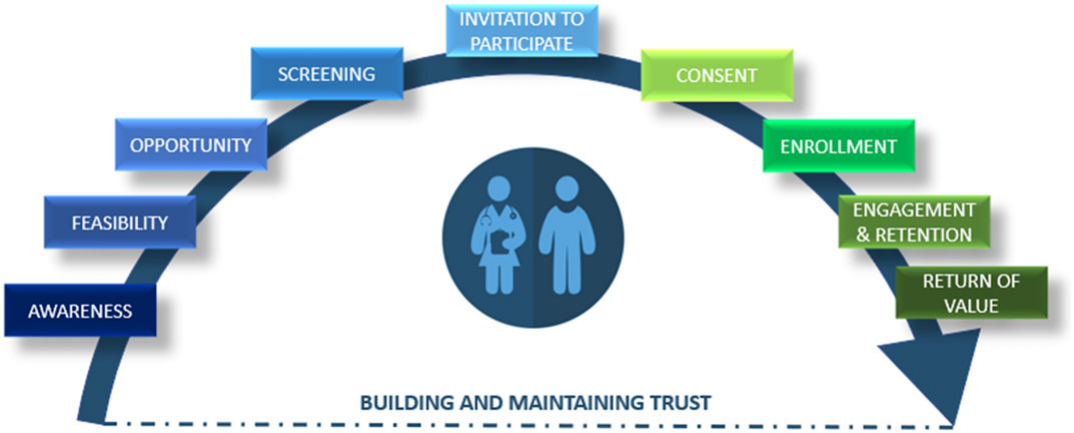
Recruitment Innovation Center continuum of participant recruitment efforts.

A guiding goal of the RIC is to shift the perception of recruitment as a one-time activity to that of an ongoing, collaborative effort across the continuum of research, from promoting awareness of research opportunities through dissemination of results and continued relationship-building (Fig. 2). The RIC focuses on building a spectrum of resources to support and integrate each component of this continuum.

### General Awareness

Promotion of clinical trials increases awareness of opportunities for research participation related to an individual’s personal needs. The RIC has worked to increase clinical trial awareness nationally with Trials Today [10], a novel public-facing interface that helps patients and families search for relevant clinical trials. Launched in 2015 and partially supported by the RIC, the site resides within the ResearchMatch volunteer platform (described later) and uses ClinicalTrials.gov data. To promote this tool, the RIC launched a national digital and print campaign in 2017 [11]. Integration between Trials Today and ResearchMatch allows volunteers to view research opportunities on a personalized ResearchMatch volunteer dashboard.

In addition, the Trials Today ‘Local’ platform allows partner institutions to display to the public the studies affiliated with their institution on a custom-branded, centrally hosted website (projecttrialstoday.org/). Trials Today Local also powers the National Covid Cohort Collaborative (N3C) clinical trial listing service [12].

### Feasibility

A major reason studies fail to meet recruitment goals is an inaccurate estimate of the number of eligible participants. To improve feasibility exploration prior to undertaking a study, the RIC developed a set of informatics resources, including cross-institutional Electronic Health Record (EHR) Cohort Assessments to determine counts of individuals with a specific diagnosis or comorbidity. The RIC has operationalized a low-tech, low-burden approach to cohort identification that has proven both feasible and adaptable across diverse types of studies and institutions, depicted and described in Supplemental Figure 1.

Because prospective sites may already be conducting trials with similar participant profiles and inclusion/exclusion criteria, the RIC has developed a Competing Trial Tool, again based on data retrieved daily from ClinicalTrials.gov. The user interface for this tool allows RIC consultants to rapidly search conditions, eligibility criteria, and expected enrollment periods for a specific trial to generate a list of known trials that might be co-enrolling at CTSA Hubs. This information is then factored into site feasibility assessment and selection. The tool exports data into a structured report that summarizes the trial, enrollment period, recruitment goals, and investigator contact information. The Competing Trial Tool is currently accessible by RIC personnel and is deployed in the context of informing specific trials during RIC consultations. Planning is underway to test and refine a public-facing tool based on experiential feedback obtained from consultations.

Another integral aspect of study feasibility is a team-based assessment of the risks, cost, time, human resources, and communications required to successfully recruit and retain participants. Often a PI does not give sufficient attention to the ways in which a study’s recruitment and retention could be impacted by these variables. Recognizing an unmet need articulated by CTSAs in our initial needs survey and substantiated in initial consultations with trialists, the RIC developed a Recruitment and Retention Plan (RRP) template to help investigators fully evaluate recruitment factors prior to grant submission. The template is comprehensive, bringing to light logistical, motivational, and behavioral barriers that could affect clinical trial recruitment and retention, and breaking them down to simplify an otherwise daunting task. In practice, completing this template can broaden the perspective of study teams, while enabling RIC consultants to generate more robust recommendations. Once complete, the RRP serves as a blueprint in determining which RIC resources could help support the study. The RIC has promulgated wide usage of this template [13], and offers assistance to PIs in completing it. The RRP can be used to meet NIH protocol guidelines, which now require an RRP.

In addition, as part of our overall comprehensive RRP, the RIC has developed a recruitment Feasibility Assessment model that may reduce barriers to clinical trial participation among diverse and rare populations. Additional components of a recruitment feasibility assessment include participant eligibility criteria such as age, gender, race, ethnicity, and health status; and potential study site environmental strengths and weaknesses such as location, competition, prior success in recruiting, and potential participant pool.

### Opportunity

The concept of opportunity refers to the participant’s ability to enroll in a specific trial. In a clinical trial consultation, the RIC guides the PI through an assessment of potential barriers that may reduce a participant’s opportunity to enroll, such as lack of clinician knowledge of the trial or willingness to refer patients to the study. The RIC suggests methods, tailored to the trial, to help minimize these obstacles.

Elements in the design of a trial, often not apparent to investigators, may unintentionally impede an individual’s opportunity to participate. Through the use of Community Engagement (CE) Studios, developed in 2009 by the Meharry-Vanderbilt Community-Engaged Research Core [14], the RIC facilitates evaluation of design elements for each trial by bringing together the study PI and multiple stakeholders (including community members) to consider the participant’s perspective when deciding such aspects as the number, duration, and frequency of study visits; study procedures; and appropriate compensation.

ResearchMatch, a disease-neutral, online service that matches study investigators with volunteers looking for trials in which to participate, is another method for increasing research participation opportunities. The platform was launched in 2009 and is funded by NIH through CTSA and RIC grants. The RIC recently supported a language translation of the ResearchMatch platform to broaden its ability to engage Spanish speakers [15].

### Screening

Participant screening is the process by which an individual is deemed eligible to join a particular study. The RIC has created a Clinical Systems Optimization (CSO) resource line to work directly with local study teams and their recruitment sites to better understand site-specific recruitment workflows, information technology (IT) capabilities, and study protocol, enabling them to provide recommendations to enhance screening efforts through EHR platforms. The CSO resource can provide investigator teams with pre-award grant language detailing how the EHR can identify eligible patients for screening and align EHR-based queries with recruitment workflows. The resource also includes documentation and guidance for leveraging bulk messaging capabilities within EHR systems to streamline the distribution of recruitment materials to potentially eligible patients. One example includes using patient portals (e.g., Epic’s MyChart) to deploy electronic bulk messaging to reach individuals potentially meeting study eligibility criteria. Retrieving patient data for research purposes requires significant policy and procedure development and implementation related to a range of issues, including regulatory requirements, ethics, participant burden, and privacy. The RIC offers guidance on using EHR workflow tools for recruitment of a targeted sample of an institution’s available population. Also provided are instructions for use, templates for patient-portal based recruitment messaging, and feedback as needed.

### Invitation to Participate

The invitation to participate in a clinical trial can be delivered via clinicians, study staff, word of mouth, mass media, social media, or printed recruitment materials such as posters, flyers, and brochures. Clinicians and study staff participating in in-person recruitment efforts need a clear understanding of the study, including eligibility criteria, study purpose, participation requirements, and potential risks and benefits. Print and electronic materials can extend a specific invitation from clinical staff to potential participants and, when appropriate, family members. The RIC provides templates for designing information sheets that detail a study for clinicians, and for designing brochures and flyers that explain requirements to potential participants. All messages and printed materials are evaluated for plain language usage, literacy level, and readability. Our continuing target is to lower the reading level of participant recruitment materials to a 6th-to-7th grade level average to accommodate potential participants with lower literacy.

Because clinician referrals are so critical to a study’s enrollment success, the RIC has developed a Referring Providers Outreach Guide, available for download in the TIN Toolbox, that provides extensive guidance to study teams in addressing provider questions and concerns about referring patients to clinical trials. The RIC has also created a Clinician Study App (CSA) template, a customizable digital resource for clinicians and coordinators to access study information (primarily on cellphones and tablets) such as inclusion/exclusion criteria, study videos, guides, and graphics (Supplemental Figure 2). The CSA allows clinicians, with a single click, to connect via email or phone call with a local study coordinator. Similarly, the RIC developed a customizable Participant Study App (PSA) template to enable rapid study information exchange with descriptive study content tailored to potential research participants. Quick Response (QR) codes can be automatically generated and printed on flyers or other print or electronic media to support smartphone utilization of PSAs. This feature allows potential participants to discreetly access study information on their phones, and also provides ‘touch-free’ access to this information during a pandemic such as COVID-19.

Traditional recruitment materials can sometimes be less effective with underrepresented groups, including minorities, who often encounter a greater array of obstacles to clinical trial participation than the non-minority population [16]. Lack of diverse participation in research can result in limited generalizability of findings and, ultimately, to health disparities [17–19]. The RIC provides support to investigators for developing specially designed recruitment strategies and materials for underrepresented groups to reduce barriers to participation. The RIC created *Faster Together, Enhancing the Recruitment of Minorities in Clinical Trials* [20], a free online training program available worldwide that teaches methods to foster recruitment and retention of racially and ethnically diverse participants in clinical research studies. The course has been shown to improve attendees’ knowledge of the material, and learners have indicated that they intend to make changes to their recruitment and retention practices as a result of their course participation [21].

### Consent

Almost one in ten clinical trial participants finds the informed consent form difficult to understand [22]. Providing researchers with strategies to maximize communication and understanding during the consent process is another key RIC resource. STRIDE (Strengthening Translational Research in Diverse Enrollment) is a CTSA partnership with Vanderbilt University Medical Center, the University of Massachusetts Medical School, the University of Alabama at Birmingham, and the Community Campus Partnerships for Health, initiated to assist researchers in making the consent process more appropriate for participants from culturally diverse or low-literacy backgrounds [23]. STRIDE’s development of a 21-CFR Part 11 compliant eConsent platform within REDCap (Research Electronic Data Capture) [24,25] was a milestone in the consenting process [26]. The RIC has played a pivotal role in refining and disseminating this work by creating an information sheet and best practices document for the consenting process, currently available online in the TIN Toolbox [13]. Additional information on eConsent is found in the Supplemental Materials.

### Engagement and Retention

Systematic efforts toward *retention* of enrolled participants are critical to achieving timely completion of a study and collecting sufficient data to power the appropriate statistical analyses. Participants can and do withdraw from a study for myriad reasons, some of which are unavoidable, such as an inconvenient location, lack of perceived improvement in their medical condition, or side effects. Some participants, however, leave a study because of difficulty with scheduling, feeling unappreciated, or through simple forgetfulness [27].

The RIC’s development of retention resources is assisted by feedback from patients and community representatives who are willing to be embedded in study teams, take surveys, act as user-testers, participate in Community Engagement Studios, or serve on advisory panels. One emerging informatics-based engagement tool is the MyCap platform, which was developed initially by our Vanderbilt REDCap team to support patient reported outcome (PRO) data from research participants in support of longitudinal studies [28] In addition to data capture, researchers can now connect to participants through the native mobile application (iOS and Android) to schedule tasks such as surveys or assessments, share announcements, and send secure messages and reminders. MyCap thus fosters enhanced communication and a general sense of connectedness and ownership among participants. Recent RIC-sponsored development work for MyCap includes language abstraction for non-English research participants (Spanish, French, and Nepali) based on local COVID-19 related use cases.

### Return of Value

A key motivator for participating in a research study is the opportunity to receive individual results, whether or not these results are clinically relevant [29,30]. Sharing results helps researchers to demonstrate the ethical principle of respect for persons [31]. The RIC has developed and disseminated extensive guidelines, templates, and examples to assist researchers in sharing study results with research participants [13].

The RIC also conducted a national survey among likely research participants [32] and found that respondents highly value results showing the impact of their personal genetic profile on likely medication response and prediction of disease risk. In addition, they often desire information about nearby clinical trials and updates on how their data are being used. The RIC is using findings from subpopulations in this study to aid researchers in identifying opportunities to deepen participants’ experience of the value yielded by their study participation.

### Building and Maintaining Trust

A major issue affecting the entire recruitment continuum is a participant’s level of trust or mistrust in researchers and institutions. Woven through many of the RIC resources is a focus on building and maintaining trust [32,33], which can contribute to a positive relationship with research that could and should last a lifetime.

While the completion of data analysis and dissemination of results signal the technical end of a study, participants who perceive value in their contribution and who trust the researchers they have encountered will likely want to participate in future studies. Former participants can continue to interact with ResearchMatch and Trials Today for future research needs. Researchers too can continue to build trust and maintain connections with former participants by sending out newsletters or staying in contact with local cultural or disease communities.

## RIC Results

### Consultations

For each study approved for support, the RIC provides a consultation with our recruitment experts. The RIC has developed multiple resources and templates that it continues to refine, while developing new ones to meet the needs of clinical trial investigators nationwide. Any given study might receive value from the RIC across a spectrum of resources: at one end, the RIC may provide an investigator with a brief consultation that includes study-specific recommendations, templates, and guidelines that have been previously built and field-tested. Mid-spectrum, the RIC may offer a mature resource line offering or innovation in need of development or refinement. At the far end, the RIC may become deeply involved in developing a needs-based recruitment or retention solution, built around a new idea, that supports the specific trial while potentially generating a solution that is generalizable to future studies. In addition, resources such as ResearchMatch and eConsent may be used by researchers on an ad hoc basis, outside of the formal consultation structure.

The RIC has completed 173 initial recruitment consultations, 164 of which were conducted for CTSA institutions. RIC resource support was offered for 107 of the consultations, while the remainder, 66, were provided with custom recruitment and retention recommendations only. Supplemental Table 3 shows the diversity of disease areas under study. The length of a RIC consultation may vary considerably (from 2 weeks to several years), based on individual study needs and joint interests in innovation or resource service line testing.

### Milestones and Achievements

The RIC, currently in Year 5 of the grant cycle, has achieved or made significant progress on all milestones proposed in our grant submission, as defined by NCATS’ strategy to develop, demonstrate, and disseminate translational research innovations [34] (Table 1).

**Table 1.** RIC milestone achievements

Grant milestone	Developed	Demonstrated	Disseminated	Comments/examples/usage*
Establish central (or shared) portal and ‘storefront’ custom to audiences	√	√	√	TIN website TIN Toolbox: 61 resources; 27,218 total views/downloads RIC Recruitment and Retention template: 208 views/downloads COVID-19 Recruitment & Retention toolkit: 458 views/downloads** “The RIC Download” bulletin: 199 subscribers, 38% average open rate
Training and continuing education courses organized and available	√	√	√	Faster Together [20,35] Referring Providers Outreach Guide: 622 views/downloads*** Art of Recruitment eConsent [26] Best Practices 75 TIN Collaboration Webinars hosted; 331 unique institutions attending; average 61 attendees per webinar ∼400 attendees at eConsent webinar in early days of COVID-19 pandemic
Study cohort estimation methods in place	√	√	√	Distribution of EHR Cohort Assessment Resource 43 studies received EHR Cohort Assessments
Linkages with the EHR for screening, patient near-term accessibility, and clinical referral	√	√		Project work in Atypical Diabetes and COVID-19. An operational Covid-19 registry including automated EHR data integration and a multi-language two-tiered consent-to-contact workflow improved recruitment [36] Proof of Concept modeling with CDSHooks using real-time EHR data extraction paired with trial eligibility criteria through the RIC TrialsToday platform Four studies received or promised Clinical Systems Optimization
ResearchMatch growth in registrants	√	√		>151,000 volunteers, 9535 participating researchers, 180 participating institutions Marketing of platform Translation of site into Spanish [15]
eConsent shared nationally with CTSA Hubs	√	√	√	1,215,000 total transactions; 18,400 projects; 594 institutions using framework in REDCap RIC toolkit in TIN Toolbox Published eConsent results [26]
Methods for returning research results to participants in place (aggregate, individual)	√	√	√	Best practices, guidelines, templates, recommendations documents published in toolkit Published papers on return of results and return of value [37,38]
Launch national PSAs	√	√		• Trials Today PSA campaign to increase awareness
Trust instrument validated and implemented	√	√		• Trust scale developed and validated but not yet disseminated
Rapid community feedback methods	√	√		• ResearchMatch model used for implementation. Working on scalable use throughout TIN or ResearchMatch partners institutions
Trials Today and Trials Today – Local platforms	√	√	√	Trials Today [10,11] and Trials Today Local platforms launched with central instance pairing with ResearchMatch and abstracted version used by many CTSA Hubs in support of local trial listing web materials 1.6 million Trials Today page views 20 medical centers and nonprofits using Trials Today Local
Research on research studies (4 planned)	√	√	√	• Studies that include research on research include the ADAPTABLE trial [39], Financial Incentives Study, Participant Preferences on Return of Value [37,38], and Project LUNA [40]
Program tracking	√	√	√	• TIN Intranet + Hub Engagement + Expression of Interest processes are serving as primary communication platform for all TIN activities – including communication with local CTSA Hub points of contact

RIC, Recruitment Innovation Center; TIN, Trial Innovation Network; EHR, electronic health records; CTSA, Clinical and Translational Science Awards; PSA, Participant Study App; CDSHooks, Clinical Decision Support Hooks.

* Data from October 2016 through February 2021, except where noted.

** Data from February 5, 2021 to June 28, 2021.

*** Data from March 1, 2019 to June 28, 2021.

### Dissemination of Recruitment Resources

A primary aim of the RIC is to disseminate not only those recruitment innovations the RIC team has designed, field-tested, and formalized, but also those developed by the broader clinical trial community. To this end, the RIC has designed and implemented these resources: the TIN website [41], which houses the TIN Toolbox, a showcase of recruitment and retention tools and resources gathered from across the CTSA community; a Collaboration Webinar series to share recruitment expertise, methods, and best practices in study recruitment; and the ‘RIC Download,’ a bi-weekly bulletin of study recruitment news and publications that highlights the latest approaches to recruitment and best practices from across the TIN.

### RIC Vignettes: Increased Study Enrollment

The RIC, in collaboration with the Project LUNA team, helped accelerate recruitment for a randomized controlled trial evaluating the efficacy of three smoking cessation strategies in long-term smokers eligible for low-dose CT lung cancer screening. The study was awarded the Recruitment Materials resource line, which consisted of a short pilot social media campaign aimed at increasing study recruitment. During the campaign, 23 participants enrolled as a direct result of the social media messaging, compared to 11 through the study’s standard recruitment channels. The initial consultation for this study spanned about 3 months, from the introductory call through submission of the recommendations report. The social media design and implementation required two months, and the manuscript describing the project was written and accepted for publication in a 20-month timeframe [40].

Another example of accelerated study recruitment facilitated by the RIC was the TARGET-RA (Rheumatoid Arthritis) study. This study was challenged with complex eligibility criteria as well as participant burden and at the time of the consult had enrolled 23 participants. The RIC provided an in-depth consultation and a myriad of resources, some of which were piloted with this study, including the CSA, Competing Trial Tool, revamped recruitment brochures, and a social media campaign. The study was eventually able to meet their revised enrollment goal of 160 in just over 24 months, and the study team has since requested RIC involvement for a new grant to employ these same types of strategies.

Additional examples of RIC consultation recommendations found helpful by investigators are contained in the Supplemental Materials.

### RIC Metrics and Evaluation

The RIC has identified metrics for evaluating research team satisfaction as well as outcomes associated with consultation and service line resource sharing efforts. The RIC regularly measures PI satisfaction with the study-specific recruitment and retention recommendations provided in our initial consultations, and has obtained consistently high ratings (Table 2). Likewise, all researchers participating in RIC Community Engagement Studios have found them worth their time and agree that the studios improved their projects. In addition, attendees of the Collaboration Webinars report high levels of satisfaction with them.

**Table 2.** Preliminary data on satisfaction with RIC consultations and resources

	Group surveyed	Measure/statement	Score
RIC Initial Consultation^a^	Principal Investigators who completed an Initial Consultation (n = 48)	Mean satisfaction (10-point scale)	9.4
Community Engagement Studios^b^	Community Experts who participated in a Community Engagement Studio (n = 88)	“The studio was worth my time.” (5-point Likert scale)	83% strongly agree; 17% agree
		“I would participate in a studio again.” (percentage saying yes)	100%
	Researchers who were Principal Investigators for a Community Engagement Studio (n = 22)	“The studio was worth my time.” (5-point Likert scale)	90% strongly agree; 10% agree
		“The studio improved my project.” (5-point Likert scale)	50% strongly agree; 50% agree
RIC-hosted TIN Collaboration Webinars^b^	Webinar attendees (n = 1801)	Mean satisfaction rating (1 = very satisfied; 5 = very unsatisfied)	1.5

RIC, Recruitment Innovation Center; TIN, Trial Innovation Network.

a Data available from 2019 through 2020.

b Data from October 2016 through July 23, 2021.

The RIC has recently begun conducting in-depth, semi-structured quality assurance interviews with PIs receiving RIC resources for their studies, to learn about their use of the resources and any barriers or facilitators to usage they have encountered. Preliminary results suggest that lack of institutional capacity and leadership buy-in may be barriers to RIC resource usage, while perceived utility, ease of use, and potential for speedy dissemination can facilitate usage. The RIC is currently conducting interviews with PIs and study coordinators of RIC-led consultations that received resources and will be obtaining study enrollment tables to gain a larger picture of enrollment trends after implementation of RIC resources.

In addition to these qualitative interviews, Table 3 includes evaluation methods for each of the RIC’s five resource service lines (see also Fig. 1) using metrics specific to that resource. Collection of some metrics has begun, while others are in the planning stages. An overarching Evaluation Plan for the RIC program was developed using a Causal Pathway approach [42] and employing logic models to describe the anticipated relationship between RIC activities and their ultimate impacts. In a causal pathway, the models specifically articulate the inputs (resources) and outputs (deliverables), and the early, intermediate, and later anticipated impacts of the initiatives, each with associated performance measures. The RIC developed a Logic Model for each of the Specific Aims of the grant for internal use as a roadmap to determine what aspects of the team’s work to evaluate. The Causal Pathway and Logic Model are described more fully in the Supplemental Materials, and an example is provided. While the models sometimes overlap—innovation developed in one aim may feed activities of another aim—they eventually converge on the common goal of the RIC and TIN: to accelerate the completion of clinical trials with representative enrollment. As the Evaluation Plan is further refined, the RIC will continue to gather a range of outcomes data to establish best practices and will report on results.

**Table 3.** Development of standard RIC resources, usage, and plans for evaluation

	Recruitment & retention planning	Feasibility & cohort assessment	Informatics solutions	Community engagement	Recruitment materials
Identified need	Study- and site- specific recruitment and retention planning, including risk assessment and identification of potential solutions	Trans-CTSA methods to inform site selection based on expected patient counts, including phenotyping for use across multiple systems and data platforms	Assessment and advisement of pragmatic use of EHR data and workflow methods in support of local trial operations	Feedback from patients and other key stakeholders needed to support effective recruitment, retention, and return of value	Development, demonstration, and dissemination of materials and resources that are accessible and understandable to targeted research populations
Bases for decision to develop resource	RIC 2016 CTSA survey results Experience with multiple teams during early initial consultation activities Recruitment & Retention Planning became a required element of NIH grant proposals for clinical trials in 2017	RIC 2016 CTSA survey results Frequent request from research community Desire to respect autonomy of CTSA Hubs	RIC 2016 CTSA survey results Requests from research community during initial consultations	Modeled after successful and highly requested service at VUMC. Requests from research community during initial consultations	Experience with multiple teams during early initial consultation activities
Method of developing resource	Created communications infrastructure to support a trans-CTSA Recruitment & Retention Working Group. This working group helped develop short and long Recruitment & Retention Plan templates that local study teams can adapt to support their particular trial. This template is used to frame assessment and support recommendations during Initial Consultations	Initial phenotyping algorithms is jointly developed by the study PI and a RIC service line lead then tested at multiple RIC partners to ensure platform neutrality and fitness of use for the EHR data query. Distribution to the CTSA community is part of the overall TIN site selection service line	RIC Informatics Working group includes team members from the Regenstrief Institute, University of Utah, Ohio State University, Columbia University, and VUMC. Collaborators at Regenstrief created decision tools for use of EHR-based recruitment methods with guidance based on study population, disease category, and other key decision criteria	The Community Engagement Studio model was established prior to the RIC, but was utilized in new ways, including recruitment of community experts from sites across the country to inform multi-site trials	Recruitment materials, both print and digital, are developed with a combination of input from study consultations, recruitment plan strategies individualized with culturally tailored messaging for each target participant population, CE Studio and/or CAB input and guidance Engagement with marketing experts to test effectiveness of messaging and develop guides for social and digital media
Effort to develop resource	0.75 FTE to develop over 9 months	1 FTE + consultants over 6 months	1 FTE + for 6 months	CE Studio model grandfathered into RIC	1.3 FTE over 1 year
Effort to sustain resource	0.30 FTE per year for sustainability	0.45 FTE per year for sustainability	1.75 FTE per year for sustainability	1.5 FTE per year for sustainability	2.5 FTE per year for sustainability
Normal timeline to implement for an individual trial	Comprehensive, custom-written Recruitment and Retention Plans require approximately 6 weeks to complete	Approximately 8 weeks from resource consultation to report delivery	Consultation level support typically consists of 1–2 meetings with the RIC and study team. Implementation depends on site readiness and EHR system maturity. For sites with existing Health IT systems and policies tuned for research, the process requires 2–3 months to fully implement	Approximately 4–6 weeks after an initial planning meeting with study team	Development and acceptance testing requires approximately 2–4 months
Usage*	14 Recruitment and Retention plans written using new template 187 recruitment experts supported by Recruitment & Retention Working Group; 140 posts and 180 post comments (January–May 2020)	43 studies provided with EHR cohort assessment	4 studies promised MyCap support	22 CE Studios conducted for RIC studies; 186 patients and community stakeholders participating	47 studies provided with custom recruitment materials; 66 total print materials (flyers, posters, etc.); 23 digital media resources (websites, social media, etc.); 14 CSAs and PSAs, with average of 711 hits and 45 submissions to study contact
Evaluation plan	Data are collected from post-resource quality assurance interviews. The RIC will obtain confidence measures after conducting a pilot survey	Top 20% ʼmost feasibleʼ sites based on cohort data are determined. Later will assess how many of the indicated top 20% were ultimately pursued by the study team as new sites	Reviews of how many referrals originate from EHR notifications to the provider versus EHR-based messaging using modules such as MyChart	Data collection from satisfaction surveys completed by study staff and community experts. Plans are to query PIs in post-resource quality assurance interviews	Qualitative interviews assessing barriers and facilitators to implementing products and utilization. For digital products, website clicks, QR code scans, conversion rates from advertisements, and A/B testing for effective messaging and design via social media

CTSA, Clinical and Translational Science Awards; EHR, Electronic Health Records; NIH, National Institutes of Health; VUMC, Vanderbilt University Medical Center; CE, Community Engagement; CAB, Community Advisory Board; FTE, Full-Time Effort; CSA, Clinician Study App; PSA, Participant Study App; PI, Principal Investigator; QR, Quick Response.

* Data from October 2016 through February 2021. Note that while all resource lines were operational from the start, individual elements within those lines may have been developed over time.

### Limitations

A limitation of the RIC model is that studies may encounter recruitment and retention challenges unique to localities or specific populations, especially those underrepresented in research. The RIC attempts to address these challenges by applying generalizable principles while also obtaining local feedback for culturally appropriate messaging, engaging local community groups for referrals, and customizing recruitment and retention planning and recommendations for each study and targeted population.

### Next Steps and Future Plans

The RIC’s suite of resources is continually evolving as more information is gained about recruitment efficacy and best practices and as new opportunities are identified. The RIC is currently developing and testing a novel expert advice feedback mechanism that can solicit opinions of ResearchMatch volunteers on a study’s proposed protocol, recruitment/engagement plan, and desired return of value. The RIC team is also developing and piloting measures of trust and person-centeredness. Future plans for the RIC include the activation of participant compensation guidelines and preparations to evaluate their effectiveness.

The RIC is in the process of adding social media engagement and recruitment website generation to our suite of recruitment resources to increase clinical trial awareness. The RIC is also developing educational materials, including videos, to train clinicians and study staff in best practices and techniques for obtaining informed consent.

The RIC will continue to innovate in the area of informatics platforms and applied methods in support of participant recruitment and engagement. Specifically, our informatics team will refine and improve methods of creating and socializing electronic phenotyping algorithms for use across diverse health systems, and has plans to advance methods for developing and deploying workflows at the intersection of research and EHR systems. New mandates from the Office of the National Coordinator for Health Information Technology (ONC) for EHR adoption of HL7 Fast Healthcare Interoperability Resources (FHIR) standard adherence are enabling systems to receive, process, and utilize clinical data in real-time [43]. The RIC is developing new models and methods for use of these data in both REDCap and ResearchMatch platforms for use as recruitment and retention tools. Work will continue to evolve in building accessible eConsent modules and participant-facing applications (e.g., Study Applications, MyCap).

The RIC intends to continue its work in developing sustainable models for grant-supported resources, services lines, and informatics tools to ensure maximal dissemination and continuity in serving the clinical trial community.

## Conclusion

The RIC is our nation’s only federally funded center charged with catalyzing enrollment in clinical trials. To address the complex recruitment and retention challenges affecting our national trial infrastructure, the RIC has created a patient-centered suite of recruitment and engagement resources to accelerate trial completion. The RIC’s overall innovation and implementation strategy scales to support the greatest number of studies with the smallest investment and allows local sites to leverage their own capabilities to take advantage of these resources. Innovations move through a life cycle of novelty, testing, and evaluation until each one becomes the new standard or best practice. The RIC’s unique team approach continues to draw on diverse expertise to build, test, and disseminate new resources that can support recruitment on all NIH-funded studies, including those outside of the TIN. Our ongoing challenge is to keep raising the bar.

## Supplementary material

For supplementary material accompanying this paper visit http://dx.doi.org/10.1017/cts.2021.841. click here to view supplementary material
