# Supplementary material for: The Recruitment Innovation Center: Developing novel, person-centered strategies for clinical trial recruitment and retention
Source: J Clin Transl Sci. 2021 Aug 19;5(1):e194. doi: 10.1017/cts.2021.841 (PMC8634298; doi:10.1017/cts.2021.841)
Supplement: Supplementary file 1 [file S2059866121008414sup001.docx]

**SUPPLEMENTAL MATERIALS**

**Contents**

1. SUPPLEMENTAL TABLES
   1. Supplemental Table 1. Results of 2016 survey of CTSAs – Perceived usefulness of potential RIC resources
   2. Supplemental Table 2. Results of 2016 survey of CTSAs – Additional areas of recruitment need
   3. Supplemental Table 3. Disease areas represented among studies receiving RIC consultations
2. SUPPLEMENTAL FIGURES
   1. Supplemental Figure 1. EHR-based cohort assessment pipeline for site feasibility
   2. Supplemental Figure 2. Sample Clinician Study App
3. ECONSENT
4. CAUSAL PATHWAY AND LOGIC MODELS
5. ADDITIONAL RIC VIGNETTES
6. **SUPPLEMENTAL TABLES**

**Supplemental Table 1.** Results of 2016 survey of CTSAs – Perceived usefulness of potential RIC resources

| **Potential RIC Resource** | **Extremely or very useful** (n=53)  **%** |
| --- | --- |
| Tools to support social media | 75 |
| Tools to support identification of potential participants | 74 |
| Evaluation of recruitment and retention | 72 |
| Development of population-specific recruitment materials | 66 |
| Training in best practices | 64 |
| Materials to support community engagement | 64 |
| Tools to support EHR use | 64 |
| Assistance with returning study results | 60 |
| Resources for online presence for recruiting participants | 60 |
| Feasibility assessment | 53 |
| Tools to assess participant experience | 51 |
| Tools to build trust | 47 |
| Creation or revision of recruitment plans | 43 |

**Supplemental Table 2.** Results of 2016 survey of CTSAs – Additional areas of recruitment need

- Complicated trials with narrow eligibility criteria
- National trials with specific minority recruitment needs
- Undiagnosed condition trials (e.g., prediabetes, mild hypertension, depression)
- Web-based screening that can miss large populations
- Engaging vulnerable populations
- Logistics issues needing pre-trial identification

**Supplemental Table 3.** Disease areas represented among studies receiving RIC consultations

| **Disease Area** | % |
| --- | --- |
| Cardiovascular diseases | 14 |
| Infectious diseases | 8 |
| Neurology | 8 |
| Pediatric disciplines | 8 |
| Pulmonary diseases | 5 |
| Oncology | 4 |
| Diabetes | 3 |
| Gastroenterology | 3 |
| Hematology | 3 |
| Trauma | 3 |
| Asthma, allergic mechanisms | 2 |
| Behavioral medicine (clinical) | 2 |
| Health services research | 2 |
| Neurodegeneration | 2 |
| Neuroscience | 2 |
| OB-GYN | 2 |
| Pediatric, prematurity, newborn | 2 |
| Psychiatry | 1 |
| Aging | 1 |
| Audiology | 1 |
| Behavioral medicine (non-clinical) | 1 |
| Clinical pharmacology | 1 |
| Clinical trials methodology | 1 |
| Dermatology | 1 |
| Disease prevention and control | 1 |
| Endocrinology | 1 |
| Exercise physiology (clinical) | 1 |
| Genomics | 1 |
| Immunology | 1 |
| Liver diseases | 1 |
| Metabolic diseases | 1 |
| Metabolism | 1 |
| Nephrology | 1 |
| Neurodegeneration | 1 |
| Ophthalmology | 1 |
| Orthopedics | 1 |
| Otorhinolaryngology | 1 |
| Pediatric hematology | 1 |
| Preventive medicine | 1 |
| Psychiatry | 1 |
| Radiology, diagnostic | 1 |
| Rheumatology | 1 |
| Surgery | 1 |
| Trauma (non-clinical) | 1 |
| Urology | 1 |
| Vaccine development | 1 |

***Data from October 2016 through February 2021**

1. **SUPPLEMENTAL FIGURES**


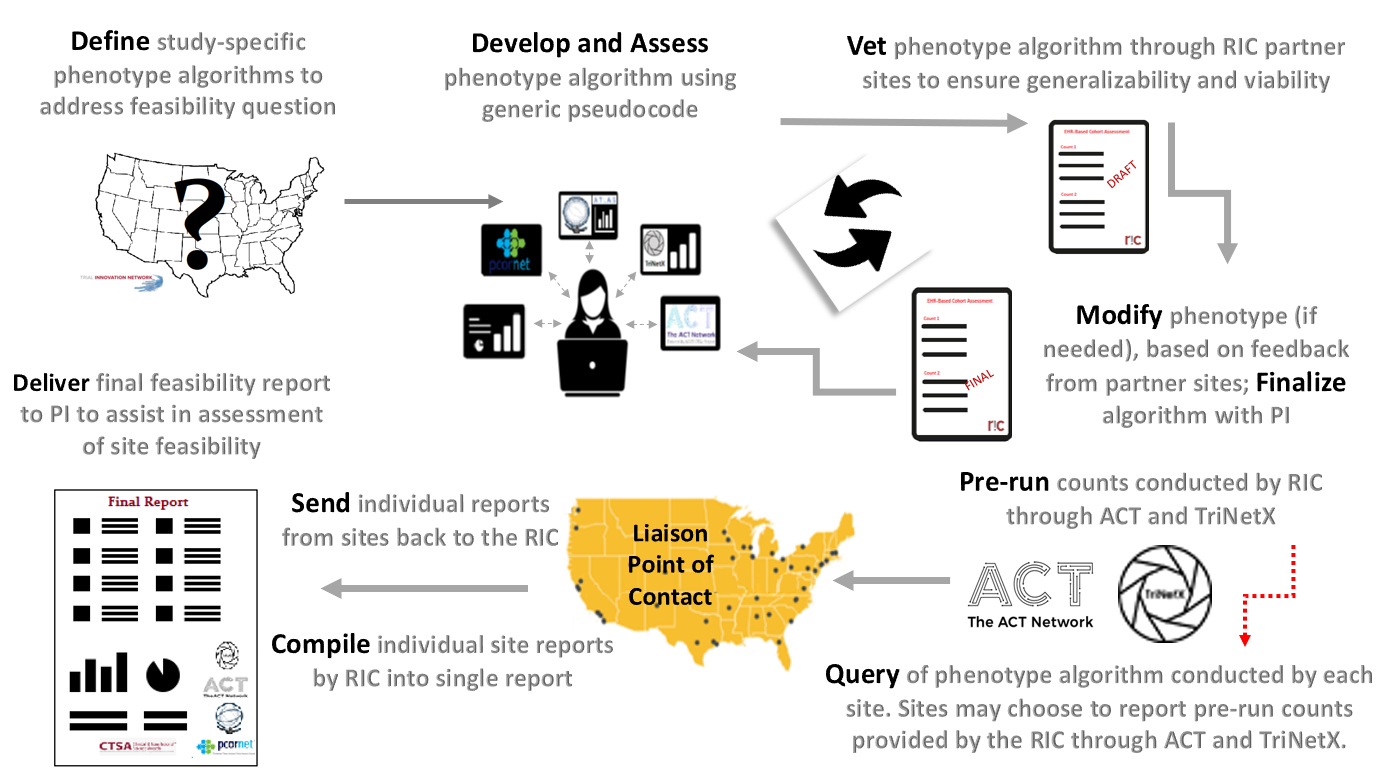


**Supplemental Figure 1.** EHR-based cohort assessment pipeline for site feasibility
 **Key:** ACT Accrual to Clinical Trials; EHR electronic health record; PI principal investigator; POC point of contact; RIC Recruitment Innovation Center

**Description of Process**: The RIC recognizes the diversity across CTSA and non-CTSA sites in sophistication of research data warehousing, availability of comprehensive data, funding for ongoing technical support and maintenance, and abilities to perform complex queries and extract data reliably. Taking these variabilities into account, we begin by defining, developing, and assessing study-specific phenotype algorithms using generic pseudocode to serve multiple data models and vetting them through our consortium partners. Results are refined as needed to enable efficient and accurate execution across varied CTSA sites. The algorithm is then pre-run through the Accrual to Clinical Trials (ACT) Network ([www.actnetwork.us/national](http://www.actnetwork.us/national)) and TriNetX ([www.trinetx.com](http://www.trinetx.com)) platforms as a final test for suitability prior to delivery to individual CTSA sites as part of a standardized TIN trial expression of interest process. Liaison points of contact (POCs) at each contacted CTSA Hub receive the request to run EHR counts at their sites using self-service tools or pass the request on to their informatics teams, then return EHR counts for the institution back to the RIC through a REDCap survey. Alternatively, sites can elect to report the pre-run counts from ACT by confirming numbers provided by the RIC or TriNetX through a permissions-based workflow developed for the RIC. Once counts are submitted, the RIC compiles results and recommendations for presentation to the investigator to facilitate informed selection of sites with the greatest potential for achieving enrollment goals.


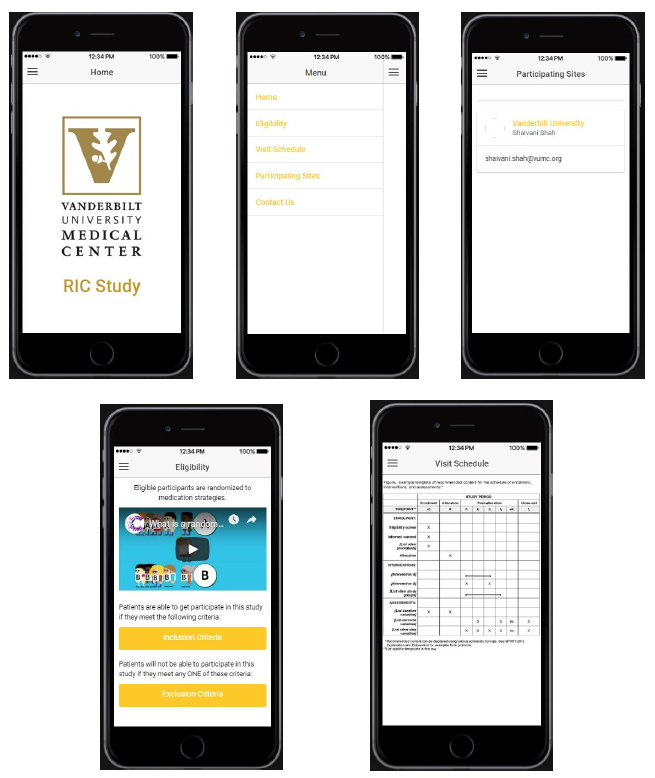


**Supplemental Figure 2**. Sample Clinician Study App, which can include: customized study graphics; menu navigation; study information such as eligibility criteria, study visit schedule, and recruitment video; and site-specific study contact with one-click access

1. **ECONSENT**

The RIC socializes the eConsent platform for TIN trials during initial and comprehensive consultations for new TIN submissions. To further promulgate use of eConsent, the RIC has supplemented STRIDE’s video library with additional videos, including ones to explain lumbar puncture and echocardiogram procedures, and two storytelling videos on the importance of clinical trials. The RIC also provides support to investigators producing their own videos for eConsent, and reviews and provides feedback on their online consent documents.

1. **CAUSAL PATHWAY AND LOGIC MODELS**

Explicitly defining the steps on performance measures in the causal pathway logic models helps to ensure that teams are aligned across initiatives, and to plan prospectively for collection of key measures. The specificity of defined causal pathway and measures enables the team to test underlying assumptions about the value and impact of various initiatives, to course-correct on the way to intended impacts, and to develop the evidence-base to support the value and dissemination of the various initiatives.

The fundamental element of the Causal Pathway is the sequence below, designed right to left, and executed left to right. In an oversimplification of design, we start with the desired ultimate impact on the right, for example, equity in research, operationalized as representative enrollment of underserved populations in research studies. The model is then developed right to left, asking at each step, “What has to happen (*before this, to the left*) for this to be true?”


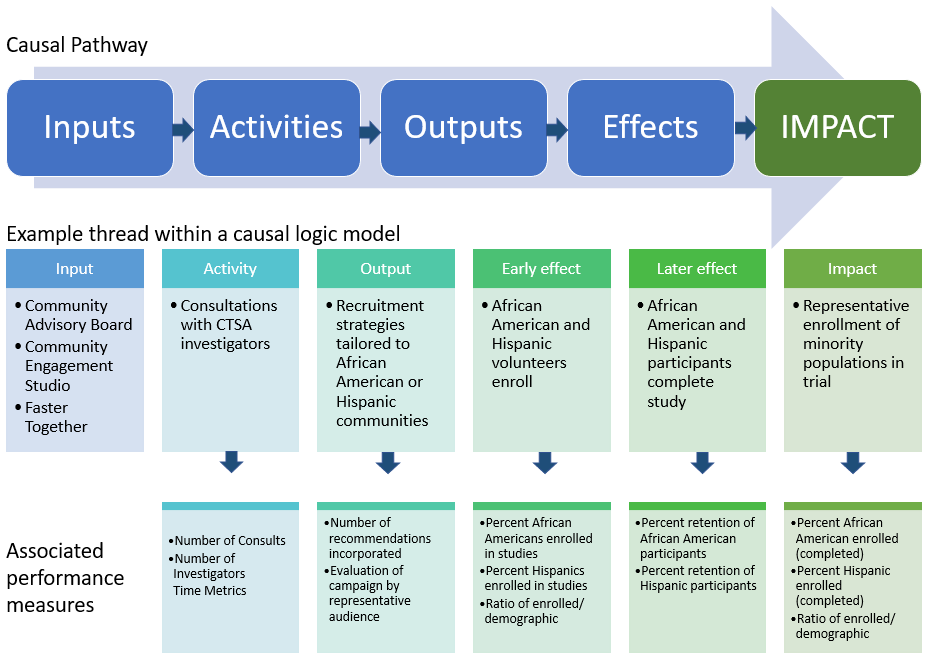


**Supplemental Figure 3. Causal Pathway approach to evaluating performance and example**

We track each specific performance measure by defining the unit of measure, how it will be collected, who is responsible for the measure, the timing, and type of analysis. Early in the causal pathway, some measures may be qualitative, in which case descriptive measures or illustrative narratives may be the elements reported. Later in the causal pathway, more quantitative measures are generally expected, such as the timeliness of accrual and percentage of the target population enrolled.

It is essential to include intermediate effects in the evaluation plan as well, for example, between the strategies for heightened awareness within a specific community and the enrollment of individuals from those communities. This intermediate effect can also be assessed with performance measures—whether clicks to websites, attendance at events, or opinion surveys. It is important data to collect because if enrollment does not in fact increase, we must then study whether it is the lack of awareness or something else interfering with the intended impact of the initiative.

The analysis of the performance data involves assessment at the project level—whether the initiatives are attaining their goals at each step, whether new challenges are identified, or new innovations inspired by outcome data, and integration at the leadership level as to the net performance across all the initiatives toward meeting the specific aims in support of TIN trials and CTSA investigators.

1. **ADDITIONAL RIC VIGNETTES**

*REACT-AF*: The Rhythm Evaluation for AntiCoagulaTion for Atrial Fibrillation (REACT-AF) trial, a multisite randomized controlled trial of patients with atrial fibrillation and other vascular risk factors, was proposed to test whether the use of a smartwatch-guided, time-delineated anticoagulant treatment could prevent strokes and reduce major bleeding events better than continuous long-term usage of anticoagulants alone. Cohort discovery led by the RIC was designed to ensure timely selection of sites with adequate enrollment capacity, including 40 CTSA sites. In the initial grant submission, reviewers expressed concern regarding participant willingness to adhere to the proposed smartwatch protocol. To address this question, the RIC conducted a survey in ResearchMatch of over 2000 potential participants. Survey results indicated that the planned data collection approach was perceived as reasonable, thereby providing a definitive answer to quell reviewers’ concerns over that aspect of study feasibility.

*MoTrPAC*: Researchers for the Molecular Transducers of Physical Activity Consortium (MoTrPAC) aimed to increase understanding of how exercise preserves tissue and organ health at the molecular level. This information would be used to help clinicians prescribe more specific exercise recommendations for their patients. The RIC conducted two CESs, one with grassroots community leaders from a low SES urban area, and the other with a diverse group of self-reported non-exercisers. Feedback from the first group was used to inform the creation of culturally tailored messaging and to identify a range of adequate compensation for the many study activities. The second group identified recruitment strategies that would be especially productive in their communities, including social media, collaboration with key community organizations, and customization of messaging for distinct groups of participants.
